# Effects of Dietary Application of the Probiotic *Lactobacillus paracasei* N1115 on Growth, Hepatic Antioxidant and Immune Biomarkers, and Intestinal Microbiota and Histology of Hybrid Sturgeon (*Acipenser baerii* ♀ × *A. schrenckii* ♂)

**DOI:** 10.1155/anu/2463494

**Published:** 2025-12-05

**Authors:** Yanchao Yang, Tianyu Liu, Ling Li, Meng Hao, Jiarou Li, Lei Li, Haiyan Liu, Baohua Zhao, Peiyu Zhang

**Affiliations:** ^1^ Laboratory of Aquatic Animal Nutrition and Ecology, College of Life Sciences, Hebei Normal University, Shijiazhuang, China, hebtu.edu.cn; ^2^ Hebei Key Laboratory of Animal Physiology, Biochemistry and Molecular Biology, College of Life Sciences, Hebei Normal University, Shijiazhuang, China, hebtu.edu.cn; ^3^ Hebei Collaborative Innovation Center for Eco-Environment, College of Life Sciences, Hebei Normal University, Shijiazhuang, China, hebtu.edu.cn

**Keywords:** antioxidant ability, growth performance, gut microbiota, *Lactobacillus paracasei* N1115, sturgeon

## Abstract

The objective of this study was to investigate the effects of incorporating *Lactobacillus paracasei* N1115 (LP N1115) into the diet on growth, hepatic antioxidant and immune biomarkers, and intestinal microbiota and histology of hybrid sturgeon. Fish with an initial body weight of 12.07 ± 0.16 g were fed four experimental diets containing 0%, 0.03%, 0.30%, and 3.0% of LP N1115 (termed as control, 0.03% LP, 0.30% LP, and 3.0% LP, respectively) twice a day (9:00 and 16:00) until apparent satiation for 56 days. The four isonitrogenous (38.72% crude protein) and isolipidic (9.89% crude lipid) diets contained viable bacteria concentrations of 0, 1.41 × 10^7^, 1.33 × 10^8^, and 1.02 × 10^9^ cfu/g diet, respectively. Each diet was randomly distributed into three tanks with 20 fish per tank (capacity: 312 L). At the end of feeding trial, whole fish was collected for body composition, and liver at postprandial 6 h was sampled for antioxidant and immune biomarkers. Mid‐duodenum was excised for morphological observation and intestinal digesta was gently squeezed for microbiota analysis. The remaining fish were exposed to 50 mg/L ammonia for 96 h post feeding trial; they were monitored every 12 h for mortality. The results indicated a significant increase in final body weight (FBW), weight gain rate (WGR), and specific growth rate (SGR) of 0.03% LP and 0.30% LP groups, along with a significant reduction in feed conversion ratio in the 0.03% LP group relative to control group (*p* < 0.05). Besides, dietary addition of 0.03% LP N1115 significantly improved hepatic activities of glutathione transferase, total superoxide dismutase (T‐SOD), and glutathione content, as well as markedly decreased hepatic contents of H_2_O_2_ and malonaldehyde. Furthermore, mRNA levels of nuclear factor‐erythroid 2‐related factor 2 (*nrf2*) and glutathione peroxidase (*gpx*) and NRF2 protein expression in the liver were significantly increased in the 0.03% LP group compared to control group (*p* < 0.05). Both villi height and muscularis thickness in the duodenum reached their maxima in the 0.03% LP group and declined at dietary probiotic levels beyond 0.03% (*p* < 0.05). Moreover, the composition of the intestinal microbiota was altered by the addition of 0.03% LP and characterized by an increase in the proportions of beneficial microbes (*Lactobacillus*, *Candidatus Arthromitus*, and *Bifidobacterium*) and a reduction in harmful bacteria (*Achromobacter*) at the genus level. Fish fed 0.03% LP and 0.30% LP diets had significantly higher survival rates at 96 h under ammonia stress compared to the control group (77.78% and 66.67% versus 55.56%) (*p* < 0.05). In conclusion, dietary LP N1115 supplementation at the dose of 0.03% (1.41 × 10^7^ cfu/g diet) could improve growth performance, hepatic antioxidant ability, ammonia‐resistant ability, and duodenal histology in juvenile sturgeon partly through altering gut microbiota.

## 1. Introduction

The hybrid sturgeon (*Acipenser baerii* ♀ × *A. schrenckii* ♂) is recognized as a highly valuable species in aquaculture due to its rapid growth rate, robust resistance against stress, and disease [1], and its boneless and flavorful flesh. Under intensive aquaculture, sturgeons frequently encountered environmental stressors such as deteriorated water quality and fluctuations in water temperature [2]. Beyond these unfavorable rearing conditions, many sturgeon farms have implemented low‐quality, high‐energy diets and overfeeding practices [3]. Such adverse practices may lead to severe oxidative stress responses in fish body, compromised overall health, and an eventual increased incidence of disease outbreaks [4]. Therefore, effective fish health management is crucial for the success of aquaculture [5]. In light of the global prohibition of certain antibiotics, aquaculture practitioners tend to utilize the probiotics to strengthen host’s health in the aquaculture operations [6]. Probiotics are defined as live microorganisms that, when supplemented into diets, could confer health benefits on animals through amending intestinal microbial population [7].

Among the probiotics, lactic acid bacteria (LAB) have attracted significant attention within both aquaculture academia and industry in recent decades [8, 9]. Certain LAB species could be applied as feed supplements for farmed animals, in accordance with Chinese Ministry of Agriculture (2013) feed additives catalogue [10], owing to their growth‐promoting and health‐enhancing functionalities. It is well documented that the efficacy of probiotics in animals is highly species‐ and strain‐specific [11]. *Lactobacillus paracasei* N1115 (LP N1115), a novel probiotic strain isolated from dairy products, exhibits strong tolerance to gastric acid (pH 2) and bile salts [12], as well as adhesive property to enterocytes [13], which facilitates its ability to reach and colonize the gut. And several studies on the efficacy of this probiotic strain have been conducted in mammals. Hu et al. [14] demonstrated that LP N1115 supplementation remarkably improved liver function and reduced inflammatory factors in hepatitis B cirrhosis patients, as well as altered intestinal microbiota composition. Moreover, the synergic application of LP N1115 and fructooligosaccharides has been shown to enhance intestinal health in mice by improving gut barrier integrity [15]. Additionally, the use of heat‐inactivated LP N1115 effectively alleviated antibiotic‐induced dysbiosis in the intestinal microbiota [16]. Regarding effectiveness of probiotics, some LABs have also been proven to improve antioxidant enzymes activities in fish besides modulating gut microbiota and improving gut barrier function. Optimal inclusion of *Pediococcus acidilactici* in zebrafish (*Danio rerio*) diet significantly improved hepatic activities of superoxide dismutase, catalase (CAT), glutathione peroxidase (GPX), and glutathione reductase [17]. Similar findings were also found in rainbow trout (*Oncorhynchus mykiss*) [18], common carp (*Cyprinus carpio*) [19], crucian carp (*Carassius auratus*) [20]. Therefore, LP N1115 may hold promise as a health‐promoting additive in fish feeds because of its multifunctional probiotic properties.

Limited research has been performed to evaluate the efficacy of incorporating *L. paracasei* into fish diets. In rainbow trout (*O. mykiss*), the addition of *L. paracasei* subsp. *tolerans* at a concentration of 10^6^ CFU/g diet led to a significant improvement in growth performance and feed utilization [21]. Similarly, the application of *L. paracasei* I61‐27 b at 10^6^–10^9^ CFU/g diet boosted growth performance, skin mucus and serum immunities, and disease resistance against *Streptococcus agalactiae* in Nile tilapia (*Oreochromis niloticus*) [22]. These findings corroborated the beneficial effects of this probiotic on fish health. It is also worthwhile noting that the influence of *Lactobacilli* is strain‐specific and depend on its population in the fish’s digestive tract [9]. To the best of our knowledge, there is currently no published data available regarding the application of LP N1115 as a potential probiotic in sturgeon diet. Given the importance of health management in sturgeon aquaculture and the potential benefits of this probiotic, the present study aims to investigate the effects of dietary administration of LP N1115 at varying doses (0, 1.41 × 10^7^, 1.33 × 10^8^, and 1.02 × 10^9^ cfu/g diet) on growth performance, as well as hepatic and intestinal health in juvenile hybrid sturgeon. The outcomes of this research are expected to enhance the understanding of LP N1115’s probiotic effects and provide a scientific foundation for its application in sturgeon aquaculture.

## 2. Methods and Materials

### 2.1. Animal Ethics Statement

The animal experiments of sturgeons in the present study adhered strictly to the guidelines of the Institutional Animal Care and Use Committee of Hebei Normal University (198017, Shijiazhuang, Hebei, China).

### 2.2. Preparation of Experimental Diets

The probiotic used in the present study is cheese‐derived LP N1115, with a concentration of viable cells at 2.1 × 10^11^ cfu/g powder. This probiotic was provided by Shijiazhuang Junlebao Dairy Co. Ltd. (Hebei, China).

Based on a previous review on the nutritional requirements of sturgeons [23], a practical basal diet formula was designed (Table 1). The basal diet formulation incorporated multiple protein sources, including animal‐derived proteins (Russian white fishmeal, American chicken meal, and squid liver powder), plant‐derived proteins (extruded soybean meal, rapeseed meal, wheat gluten meal, and cottonseed protein concentrate), and single cell protein (beer yeast powder). Soybean oil and soybean lecithin were the primary lipid sources, pregelatinized corn starch served as the primary carbohydrate source. Simultaneously, three iso‐nitrogenous and iso‐lipidic probiotic diets formulation were also designed in Table 1. During the diet manufacturing process, all ingredients, except for the probiotics and soybean oil, were weighed based on the percentage quantities in Table 1. They were then finely ground to pass through an 80‐mesh sieve and thoroughly blended with soybean oil. Subsequently, an additional 23% of distilled water was added and thoroughly mixed with the mixture using an electric blender for ~15 min to form a dough. This dough was then pelletized using a laboratory feed machine (Youyi Machinery Factory, Shandong, China) to produce pellets with a diameter of 2 mm. The pellets were then dried at 50°C in a ventilated oven to achieve a moisture content of ~8%. Afterwards, the diets were cooled to room temperature, packaged in ziplock bags, and stored at −20°C in a refrigerator until use. Given that high extrusion temperature during the pelleting process may cause deactivation of probiotic cells. Therefore, probiotics were incorporated post‐pelleting via a spraying method. Varying concentrations (0.03%, 0.3%, and 3.0%) of probiotic powder were suspended in distilled water and sprayed on the surface of the manufactured pellet. Following sufficient absorption, the pellets were then dried at 37°C in an oven for 12 h and preserved in sealed bags in a freezer at −20°C. To check the storage effect of the probiotic diets at −20°C, viable cell counts in the three diets were detected on day 0, day 7 and day 14 using bacterial spread plate technique. The viable cell counts for the 0.03% LP, 0.30% LP and 3.0% LP diets were as follows: day 0 (1.41 × 10^7^, 1.33 × 10^8^, and 1.02 × 10^9^ cfu/g diet), day 7 (1.40 × 10^7^, 1.22 × 10^8^, and 1.01 × 10^9^ cfu/g diet), day 14 (1.32 × 10^7^, 1.16 × 10^8^, and 0.97 × 10^9^ cfu/g diet). These results indicate that probiotic viability remained largely stable over a 2‐week period at −20°C. Consequently, probiotic diets were prepared biweekly and stored at −20°C.

**Table 1:** Diet formula and proximate chemical composition of the four experimental diets (% dry matter basis).

Ingredients	Control	0.03% LP	0.30% LP	3.0% LP
Russian white fishmeal^a^	14.55	14.55	14.55	14.55
American chicken meal^b^	9.70	9.70	9.70	9.70
Squid liver powder^c^	6.79	6.79	6.79	6.79
Extruded soybean meal^d^	17.19	17.19	17.19	17.19
Rapeseed meal^e^	7.76	7.76	7.76	7.76
Wheat gluten meal^f^	6.31	6.31	6.31	6.31
Cottonseed protein concentrate^g^	2.91	2.91	2.91	2.91
Beer yeast powder^h^	4.95	4.95	4.95	4.95
α‐corn starch^i^	16.49	16.49	16.49	16.49
Limestone	1.00	1.00	1.00	1.00
Sodium chloride	1.00	1.00	1.00	1.00
Magnesium sulfate	0.30	0.30	0.30	0.30
Potassium chloride	0.20	0.20	0.20	0.20
Citric acid	0.50	0.50	0.50	0.50
Mineral and vitamin premix^j^	2.50	2.50	2.50	2.50
Lysine	0.20	0.20	0.20	0.20
Methionine	0.15	0.15	0.15	0.15
Betaine	0.10	0.10	0.10	0.10
Choline chloride^k^	0.20	0.20	0.20	0.20
Taurine	0.40	0.40	0.40	0.40
Soybean oil	3.00	3.00	3.00	3.00
Soybean lecithin	0.80	0.80	0.80	0.80
Microcrystalline cellulose	3.00	2.97	2.70	0.00
*Lactobacillus paracasei* N1115	0.00	0.03	0.30	3.00
Sum	100.00	100.00	100.00	100.00
Chemical composition of pellets (% dry weight)				
Moisture	7.80	8.06	7.92	8.45
Crude protein	38.06	39.10	38.65	39.08
Crude lipid	9.87	9.41	9.83	10.45
Ash	11.68	11.49	11.50	11.66

*Note:* Control, 0.03% LP, 0.30% LP, 3.0% LP are designated as experimental diets containing 0, 0.03%, 0.30%, 3.0% LP N1115, respectively.

^a^Russian white fishmeal: JSC Okeanrybflot, Petropavlovsk Kamchatsky, Russia.

^b^American chicken meal: Fieldale Farms Corporation, USA.

^c^Squid liver powder: Cangzhou Haitong Bio‐Feed Co. Ltd., Hebei, China.

^d^Extruded soybean meal: Hebei Haitai Technology Co. Ltd., Hebei, China.

^e^Rapeseed meal: Coland Feed Co. Ltd., Wuhan, Hubei, China.

^f^Wheat gluten meal: Kaifeng Shangdu Co. Ltd., Henan, China.

^g^Cottonseed protein concentrate: Handan Chenguang Biotechnology Co. Ltd., Hebei, China.

^h^Beer yeast powder: Hebei Ruiqi Biotechnology Co. Ltd., Hebei, China.

^i^α‐corn starch: Ningjin Jiahe Energy Saving Material Co. Ltd., Shandong, China.

^j^1 kg of premix provides the following vitamins: retinyl acetate, 11.2 mg; cholecalciferol, 1.68 mg; α‐tocopheryl acetate, 2000 mg; menadione nicotinamide bisulfite (MSN), 982.8 mg; thiamin hydrochloride, 600 mg; riboflavin, 240 mg; pyridoxine hydrochloride, 2400 mg; cyanocobalamin, 2 mg; myo‐inositol, 13,444 mg; calcium‐D, pantothenate; 400 mg; niacin, 1200 mg; folic acid, 140 mg; biotin, 20 mg; ascorhyl acetate, 8000 mg; and the premix also incorporated the following minerals: CuSO_4_·5H_2_O, 1670.4 mg; FeSO_4_·7H_2_O, 20,000 mg; MnSO_4_·H_2_O, 1647.27 mg; ZnSO_4_·7H_2_O, 5317 mg; CoCl_2_·6H_2_O, 240.03 mg; ammonium molybdate, 242.03 mg; KI, 235.3 mg; Na_2_SeO_3_, 90.13 mg. All micronutrients were diluted with zeolite to 1 kg.

^k^Choline chloride: 50% choline chloride and 50% silicon dioxide.

Afterwards, the proximate chemical composition (dry matter, crude protein, crude lipid, and ash) of the experimental diets was analyzed according to the protocols of AOAC [24]. Briefly, moisture content was determined by drying samples at 105°C for 3 h (method 934.01). Crude protein content (N × 6.25) was examined using the Kjeldahl method with a Kjeltec 8420 Analyzer (FOSS, Denmark) (method 954.01). Crude lipid content was analyzed by Soxhlet extraction method using petroleum ether (method 920.39). Ash content was determined by incinerating the samples in a muffle furnace at 550°C for 3 h (method 942.05). The proximate chemical composition of experimental diets is presented in Table 1.

### 2.3. Experimental Fish and Rearing Management Regime

Three hundred (300) healthy hybrid sturgeon juveniles with a similar weight of about 8 g were obtained from the Jingxing Liyuan sturgeon base in Shijiazhuang, China. They were transported in two large plastic carrying buckets equipped with an electric air pump to the aquaculture system at the College of Life Sciences of Hebei Normal University, where the feeding trial took place. After balancing water temperature and sterilizing with 25.6 g/kg of saline water, fish were acclimatized to the culture conditions and feeding regime over a 2‐week period. During this adaption period, fish were hand‐fed the basal diet twice daily (at 9:00 and 16:00) until satiated.

Following acclimatization, the fish were fasted for 24 h. Three samples of four fish were taken for analysis of initial whole‐body chemical composition. Thereafter, sturgeon juveniles were counted into groups of 20 fish, bulk‐weighed (average weight 12.08 g), and randomly distributed amongst 12 rectangular 312 L plastic tanks. An air stone was fitted inside each tank and ran for 24 h/day for a well oxygenation in water. Each diet was offered in excess to fish in three tanks twice per day (9:00 and 16:00) for 56 days. Each meal lasted 30 min. Unconsumed food was collected by siphoning 30 min after each meal, dried overnight at 105°C, and weighed to enable reliable calculations of food intake to be made. Hebei Normal University utility water was used as the freshwater source for fish rearing, with approximately one‐third of the water in each tank being exchanged daily. Throughout the feeding trial, the fish were exposed to a 12L:12D photoperiod with artificial light switched on at 08:00 and off at 20:00, illumination intensity at the water surface in the middle of the tank was 29.8–41.4 Lx. Water temperature (20.7–22.8°C), pH (7.6–7.9), ammonia concentration (0.4–0.6 mg/L), nitrite level (0.01–0.05 mg/L), and dissolved oxygen level (4–5 mg/L) were weekly monitored and recorded.

### 2.4. Sample Collection

At the end of the 3rd, 6th, and 8th week of the growth trial the fish in each tank were counted and bulk‐weighed and growth performance parameters were calculated. 2 days after the bulk‐weighing in the 8th week, the fish were hand‐fed to apparent satiety and sampling started 6 h later. Three fish were taken from each tank, anesthetized using eugenol (60 mg/L), and body weight and body length were measured individually for calculation of condition factor (CF). Thereafter the fish were bled, killed, and dissected. The viscera were removed and weighed for calculation of the viscero‐somatic index (VSI). The liver was separated from the viscera and weighed for calculation of the hepato‐somatic index (HSI). Subsequently, the liver was snap‐frozen in liquid nitrogen and stored until analyzed for antioxidant and immunity biomarkers. The gut was separated from the remaining viscera, and the intestinal digesta was gently squeezed out using sterile tweezers. The digesta was frozen in liquid nitrogen and stored at −80°C for subsequent analysis of gut microbiota. About 0.5 cm of the mid‐duodenum was excised, and fixed in 4% paraformaldehyde, for later histological analysis. Finally, an additional three fish were randomly chosen for measurement of CF and organ indices (VSI and HSI). Subsequently, the visceral mass was returned to the abdominal cavity, and the entire fish was stored at −20°C for further analysis of whole‐body chemical composition.

### 2.5. Ammonia Stress

Right after the sampling for the feeding trial, the remaining fish (*n* = 14) in each tank were subjected to acute ammonia stress. The ammonia stress test was performed within the same tanks as used during the growth trial. The ammonia concentration used in this study was set at 50 mg/L [25], achieved by adding a mother solution of NH_4_Cl (10 g/L). The ammonia concentration was monitored and adjusted every 6 h. The number of dead fish per tank was recorded every 12 h during 96 h. The Kaplan Meier survival plot was drawn with GraphPad Prism 7.0.0 version as reported in [26].

### 2.6. Biochemical Analysis

The proximate composition analyses in the initial and final fish samples were consistent with diet samples. Two of the three liver samples from each tank were randomly chosen and used for antioxidant and immunity analyses. Around 100 mg of liver tissues were homogenized in ice‐cold saline solution (1:9 w/v ratio) with steel beads using an automatic multiple‐sample tissue homogenizer (JXFSTPRP‐32, Shanghai Jingxin Industrial Development Co., Ltd., Shanghai, China). Thereafter, the homogenates were centrifuged (2500 rpm, 10 min, 4°C) and the supernatants were collected for further analyses. The contents of glutathione (GSH) and malondialdehyde (MDA), as well as enzymatic activities of GPX, glutathione S‐transferase (GST), total superoxide dismutase (T‐SOD), CAT, peroxidase (POD), acid phosphatase (ACP), and alkaline phosphatase (ALP) in the liver samples were tested using commercial kits per the manufacturer’s instructions (Nanjing Jiancheng Bioengineering Institute, Nanjing, Jiangsu, China). The hepatic hydrogen peroxide (H_2_O_2_) content was measured according to the specifications of commercial kit from Beijing Solarbio Science & Technology Co., Ltd. The total antioxidant capacity (TAC) was detected using a kit from Sangon Biotech (Shanghai, China).

### 2.7. Determination of Duodenal Histology

Two fixed duodenum samples from each replicate were randomly selected, and the H&E staining operation was conducted by Wuhan Servicebio Technology Co., Ltd. The protocol was briefly delineated as follows. The fixed sample fragments underwent hydration processes with a graded series of alcohol solutions and xylene, and then were embedded using paraffin. Tissue blocks were sectioned with ~4 µm thickness using a Leica RM2016 automatic rotary microtome (Shanghai, China). A total of six tissue sections per treatment (one section per fish) were stained with hematoxylin and eosin (H&E). Morphometric evaluation of the tissues was performed using a fluorescence microscope imager equipped with a Leica camera (Leica DFC9000). The villus height (µm) and muscular layer thickness (µm) were measured using the ImageJ software (https://imagej.net/ij), and measurement method is consistent with that of Bellinate et al. [27].

### 2.8. Quantitative PCR to Measure Antioxidant and Immune‐Related Genes in the Liver

Two of the three liver samples from each tank were randomly chosen and subjected to RNA isolation using the Trizol method. The quality and concentration of RNA were evaluated by agarose gel electrophoresis and NanoDrop 2000 ultramicro‐spectrophotometry (ThermoScientific, USA). Then the complementary DNA (cDNA) was synthesized using a First Strand Synthesis Kit (ThermoScientific, USA). Quantitative real‐time PCR (RT‐PCR) was performed on a quantitative thermal cycler (CFX96 Real‐Time PCR System, USA) using 2 × TransStart Top Green qPCR SuperMix (TransGen Biotech, Beijing, China) containing 10 μM gene specific primer. The amplification was performed in a 20 μL reaction volume comprising 10 μL of qPCR SuperMix, 0.4 μL of each primer, 2 μL of cDNA template, and 7.2 μL of nuclease‐free water. The thermal cycling protocol for PCR consisted of initial denaturation at 94°C for 30 s, followed by 48 cycles at 94°C for 5 s, annealing and extension for 30 s. Two technical replicates were performed for each sample in the qPCR analysis. The 18 s‐rRNA gene were used as the housekeeping gene in this study and the detailed information and amplification condition are provided in Table 2. The relative transcriptional level of the target genes was computed according to 2^−ΔΔCt^ method [28].

**Table 2:** Primer sequence, product size, and accession number of target genes used for real‐time PCR (RT‐PCR).

Genes	Primer sequence 5′‐3′	Amplicon size (bp)	Accession number
*18s-rRNA*	F: CCGCTTTGGTGACTCTGGAT	193	AY544132.1
	R: CTTGGATGTGGTAGCCGTTTC		
*nrf2*	F: CTGGCAGAGTCGTTCC	424	XM_034045119.2
	R: TTAGGTTCTCGGGTGG		
*keap1*	F: TGCGTTGCGAGTCTGA	139	XM_034002868.2
	R: AGCTGGGTCTGGAGGAA		
*gpx*	F: CCCTTCACTGTTCTCG	358	XM_034034142.3
	R: AATCCTCCTCCTGGTC		
*gr*	F: GAGTGGAGGTAAATGG	492	XM_034031269.2
	R: GTGCGTCTAGGTTGAG		
*tlr5*	F: AGAGGACACAGTCGCTAAGT	246	XM_034017534.3
	R: TCAGCCGCTCTATGTTGTTG		
*tak1*	F: AGTCGGTCCTCTTCCTC	123	XM_059025842.1
	R: AATCTCGGCATTCTCAA		
*il-8*	F: TATCAATGCTGCTCCT	113	XM_034036032.3
	R: GTGGTCCTTCTGGTGT		
*tnf-α*	F: AACAACTGGCGAGCAA	148	XM_059010883.1
	R: CTGGGCACCACAATCT		
*il-1*β	F: GAGAAGATGAAGAGACCGCA	104	XM_059020519.1
	R: AGGATCACGTGCTCTTCATT		
*tgf-β*	F: GCAGCTGTTCTTCAACATGT	141	XM_033992899.3
	R: GTGCCCTTGTACAGCTCTAT		

*Note*: *nrf2*, nuclear factor erythroid 2‐related factor 2; *keap1*, kelch like ECH associated protein 1.

Abbreviations: *gpx*, glutathione peroxidase; *gr*, glutathione reductase; *il-8*, interleukin‐8; *il-1*β, interleukin‐1β; *tak1*, *tgf-β*‐activated kinase 1; *tgf-β*, transforming growth factor‐β; *tlr5*, toll‐like receptors 5; *tnf-α*, tumor necrosis factor α.

### 2.9. Western Blot Analysis

The procedures of protein extraction and Western blot were in line with our previous study [29]. One of three liver samples from each tank (*n* = 3 for each treatment) was randomly selected for Western blot analysis. Briefly, protein extraction from the liver was performed, followed by SDS‐PAGE electrophoresis and membrane transfer. Afterwards, the membrane was incubated with primary antibody overnight on a shaker at 4°C. The primary polyclonal antibodies used in the present study included rabbit antibodies against nuclear factor erythroid 2‐related factor 2 (NRF2) (1:750, Cat#WL02135, Wanleibio) and the reference protein GAPDH (1:2000, Cat#GB15004, Servicebio). Following membrane washing, anti‐rabbit second antibody was incubated at room temperature for 1 h. After washing with TBST, chemiluminescence was performed using an Ultrasensitive ECL Chemiluminescence kit (Biosharp, China). The protein bands on the membrane were captured and analyzed by ImageJ software.

### 2.10. Intestinal Microbiota Analysis

The intestinal digesta from three fish per treatment (*n* = 3 per treatment) were subjected to genomic DNA extraction. Total genomic DNA of digesta samples was extracted in a sterile environment using the FastDNA Spin Kit for Soil (MP Biomedicals, USA) following the manufacturer’s protocol. The quantity and quality of genomic DNA were assessed by NanoDrop 2000 ultramicro‐spectrophotometry and agarose gel electrophoresis, respectively. The V3‐V4 hypervariable region of the bacterial 16 S rRNA gene was amplified using the primer pair: 338 F (5′‐ACTCCTACGGGAGGCAGCAG‐3′) and 806 R (5′‐ GGACTACHVGGGTWTCTAAT‐3′). After purification and quantification, the PCR products were sequenced on the Illlumina Nextseq 2000 platform (Shanghai Biomedical Technology Co., Ltd.). The sequences were then quality filtered, denoised, merged, and chimeric sequences were removed through UCHIME algorithm and Gold database [30]. The effective sequences were clustered to OUT (operational taxonomic unit) based on 97% similarity, and representative sequences were screened and annotated. Alpha diversity was measured using the Sob, Chao, Ace, Shannon, and Simpson parameters, and statistical differences were analyzed using one‐way ANOVA (*p* < 0.05). Beta diversity was assessed utilizing principal coordinate analysis (PCoA) based on weighted Unifrac distance metric. Venn diagrams and 3D‐PCoA plots were created on the platform of MicrobiomeAnalyst 2.0 (https://www.microbiomeanalyst.ca/).

### 2.11. Calculations and Statistical Analyses

#### 2.11.1. Calculations of Growth

Feeding rate, FR% BW/d=100×total feed intake/days/IBW+ FBW/2,

Weight gain rate, WGR%=100×FBW−IBW/IBW,

Specific growth rate, SGR%=100×lnFBW−lnIBW/days,

Feed conversion ratio, FCR= dry feed intake/wet body weight gain,

Protein efficiency ratio, PER= wet body weight gain/dry protein intake,

Protein retention efficiency, PRE%=100×final fish protein weight−initial fish protein weight/protein intake,

Condition factor, CF=100×body weight/body length3,

Hepatosomatic index, HSI%=100×liver weight/FBW,

Viscerosomatic index, VSI%=100×viscera weight/FBW,

Survival rate, SR%=final fish number/initial fish number×100,

wherein FBW and IBW were final body weight and initial body weight, respectively.

#### 2.11.2. Statistical Analyses

Considering that the tank, rather than individual fish, was the independent experimental unit for data presentation and analysis. Morphological parameters (HSI, VSI, and CF) were individually measured in six fish per tank, and their mean values for each tank were used in statistical analyses. Three fish per tank were pooled as a sub‐sample, and three sub‐samples were analyzed for fish‐body chemical composition. The hepatic physiological indices, as well as immune response and antioxidant ability‐related genes expression in liver of two fish per tank were measured, and their mean values were utilized for statistical evaluation. Additionally, duodenal villi height and muscular layer thickness of one section per fish were quantified, and their mean values were used for further analysis.

The data were analyzed using SPSS software (IBM SPSS Statistics 19.0, IBM, USA) presented as mean ± standard error (SE). Shapiro–Wilk and Levene’s test was utilized to assess normality and homogeneity of data. All data were analyzed by one‐way analysis of variance (one‐way ANOVA), and Tukey’s test was used to compare the mean values between individual treatments when overall differences were significant (*p* < 0.05).

## 3. Results

### 3.1. Growth Performance, Feed Utilization, and Body Morphology

Growth performance, feed utilization, and body morphology of juvenile sturgeon fed four experimental diets containing different doses of LP N1115 for 8 weeks are presented in Table 3. The mean body weight of the sturgeon at the 3rd week and 6th week did not differ among groups (*p* < 0.05), while it reached significant level at 8th week and it was significantly higher in 0.03% LP and 0.3% LP groups compared with that in the control group (*p* = 0.003). The WGR and SGR were also statistically improved by dietary supplementation of this probiotic at the doses of 0.03% and 0.3% relative to the control group (*p* < 0.01). In addition, a significant decrease in FCR in 0.03% LP group, as well as a significant reduction in VSI in 0.03% and 0.3% LP groups were found when comparing with counterparts in the control group (*p* < 0.05). No significant changes were noted among groups in terms of FR, PER, PRE, HSI, CF, and SR (*p* > 0.05).

**Table 3:** Growth parameters, nutrient utilization, body morphology in juvenile hybrid sturgeon fed four experimental diets containing different doses of *L. paracasei* N1115 for 8 weeks.

Parameters	Control	0.03% LP	0.3% LP	3.0% LP	*p* Value
IBW (g/fish)	12.05 ± 0.12	12.05 ± 0.12	12.11 ± 0.10	12.07 ± 0.12	0.974
FR (%/d)	2.19 ± 0.01	2.10 ± 0.01	2.13 ± 0.07	2.18 ± 0.01	0.290
BW 3rd week (g/fish)	34.38 ± 1.50	37.34 ± 1.84	37.42 ± 0.85	35.80 ± 1.27	0.421
BW 6th week (g/fish)	79.09 ± 2.58	85.62 ± 2.06	83.09 ± 3.83	79.73 ± 2.81	0.400
FBW 8th week (g/fish)	91.02 ± 2.63^a^	107.71 ± 1.47^c^	102.22 ± 1.32^bc^	97.58 ± 2.54^ab^	0.003
WGR (%)	655.87 ± 23.40^a^	794.21 ± 3.76^c^	743.98 ± 4.64^bc^	709.00 ± 25.67^ab^	0.003
SGR (%/d)	3.61 ± 0.06^a^	3.91 ± 0.01^b^	3.81 ± 0.01^b^	3.73 ± 0.06^ab^	0.004
FCR	0.80 ± 0.004^b^	0.74 ± 0.003^a^	0.76 ± 0.023^ab^	0.77 ± 0.004^ab^	0.021
PER	3.29 ± 0.02	3.47 ± 0.01	3.43 ± 0.10	3.27 ± 0.02	0.058
PRE (%)	41.27 ± 0.36	45.30 ± 0.39	44.70 ± 2.01	42.34 ± 1.00	0.112
HSI (%)	3.21 ± 0.05	3.41 ± 0.20	3.13 ± 0.06	3.41 ± 0.02	0.235
VSI (%)	13.05 ± 0.17^c^	11.88 ± 0.04^ab^	11.77 ± 0.35^a^	12.86 ± 0.50^bc^	0.043
CF	1.47 ± 0.05	1.61 ± 0.04	1.47 ± 0.05	1.56 ± 0.05	0.157
SR (%)	100.00 ± 0.00	100.00 ± 0.00	100.00 ± 0.00	100.00 ± 0.00	—

*Note:* Values were presented as means ± SE (*n* = 3). Values in the same row with different superscript letters denoted statistical differences evaluated by Tukey’s test (*p* < 0.05).

Abbreviations: BW, body weight; CF, condition factor; FBW, final body weight; FCR, feed conversion ratio; FR, feeding rate; HSI, hepatosomatic index; IBW, initial body weight; PER, protein efficiency ratio; PRE, protein retention efficiency; SGR, specific growth rate; SR, survival rate; VSI, viscerosomatic index; WGR, weight gain rate.

### 3.2. Whole‐Body Composition

The proximate chemical composition of the initial and final fish samples is shown in Table 4. There were no significant variations in the contents of moisture, crude protein, crude lipid, and ash among groups (*p* > 0.05).

**Table 4:** Whole‐fish body composition in juvenile hybrid sturgeon fed four experimental diets containing different doses of *L. paracasei* N1115 for 8 weeks (%, on wet weight basis).

Parameters	Initial fish sample	Control	0.03% LP	0.30% LP	3.0% LP	*p* Value
Moisture	85.95 ± 0.47	79.58 ± 0.05	78.67 ± 0.44	78.65 ± 0.18	78.50 ± 0.20	0.064
Crude protein	8.65 ± 0.26	12.05 ± 0.07	12.55 ± 0.14	12.52 ± 0.20	12.41 ± 0.22	0.212
Crude lipid	2.28 ± 0.28	4.51 ± 0.22	5.24 ± 0.39	4.93 ± 0.14	5.19 ± 0.28	0.283
Ash	2.51 ± 0.08	2.47 ± 0.03	2.43 ± 0.06	2.47 ± 0.09	2.52 ± 0.05	0.807

*Note:* Values were presented as means ± SE (*n* = 3).

### 3.3. Antioxidant‐Related Parameters in Liver

The impact of dietary administration of LP N1115 on hepatic antioxidant‐related parameters in sturgeon is displayed in Table 5 and Figure 1. Notably, hepatic GSH content in probiotic supplemented groups, as well as activities of GST and T‐SOD in 0.03% LP groups were significantly improved compared to control group (*p* < 0.05). Furthermore, a notable decrease in contents of H_2_O_2_ and MDA, and activities of CAT and POD in 0.03% LP group were observed relative to control group (*p* < 0.05). Regarding TAC, the 0.30% LP group achieved the highest value, followed by the 0.03% LP group and then the 3.0% LP and control groups. Moreover, hepatic mRNA levels of *nrf2* and *gpx*, as well as NRF2 protein expression were significantly up‐regulated in the 0.03% LP group compared to control group (*p* < 0.05).

**Table 5:** Hepatic antioxidant‐related parameters and immunological indices in juvenile hybrid sturgeon fed four experimental diets containing different doses of *L. paracasei* N1115 for 8 weeks.

Parameters	Control	0.03% LP	0.30% LP	3.0% LP	*p* Value
Antioxidant‐related parameters					
GSH (mg/gprot)	6.39 ± 0.56^a^	15.39 ± 0.71^c^	15.01 ± 0.27^c^	11.53 ± 0.63^b^	<0.001
GST (U/gprot)	17.89 ± 0.55^a^	29.20 ± 0.97^b^	24.69 ± 2.51^ab^	23.35 ± 1.45^ab^	0.006
GPX (U/gprot)	24.07 ± 4.60	22.50 ± 1.74	25.50 ± 1.18	24.37 ± 1.38	0.878
T‐SOD (U/mgprot)	414.98 ± 17.00^a^	601.84 ± 54.59^b^	491.79 ± 12.94^ab^	424.57 ± 37.05^a^	0.018
H_2_O_2_ (μmol/gprot)	9.66 ± 0.27^b^	6.76 ± 0.88^a^	7.71 ± 0.42^ab^	8.74 ± 0.38^ab^	0.025
CAT (U/mgprot)	41.64 ± 1.82^b^	25.86 ± 1.75^a^	29.07 ± 3.19^a^	32.98 ± 3.24^ab^	0.013
POD (U/mgprot)	36.78 ± 1.60^b^	24.23 ± 2.20^a^	27.27 ± 4.08^ab^	21.72 ± 0.99^a^	0.013
MDA (mmol/gprot)	8.78 ± 0.71^b^	4.62 ± 0.62^a^	5.99 ± 0.79^ab^	4.10 ± 0.01^a^	0.008
TAC (μmol/gprot)	84.96 ± 5.89^a^	108.38 ± 5.32^ab^	114.79 ± 5.53^b^	85.03 ± 7.23^a^	0.014
Immunological indices					
ALP (King unit/gprot)	96.32 ± 3.18^b^	67.94 ± 8.32^a^	72.66 ± 3.30^ab^	96.26 ± 5.33^b^	0.020
ACP (King unit/gprot)	56.48 ± 2.85^ab^	46.10 ± 2.27^a^	51.68 ± 0.70^ab^	62.20 ± 4.37^b^	0.017

*Note:* Values were presented as means ± SE (*n* = 3). Values in the same row with different superscript letters denoted statistical differences evaluated by Tukey’s test (*p* < 0.05).

Abbreviatons: ACP, acid phosphatase; ALP, alkaline phosphatase; CAT, catalase; GPX, glutathione peroxidase; GSH, glutathione; GST, glutathione S‐transferase; MDA, malondialdehyde; POD, peroxidase; TAC, total antioxidant capacity; T‐SOD, total superoxide dismutase.

Figure 1The effects of dietary supplementation of *L. paracasei* N1115 on the hepatic parameters associated with antioxidant ability and immune response in sturgeon. Antioxidant‐related genes expression (A); NRF2 protein expression (B); immune‐related genes expression (C). All data are shown as mean ± SE (*n* = 3). The lower cases above columns indicate significant differences among groups (*p* < 0.05).

**Figure:**
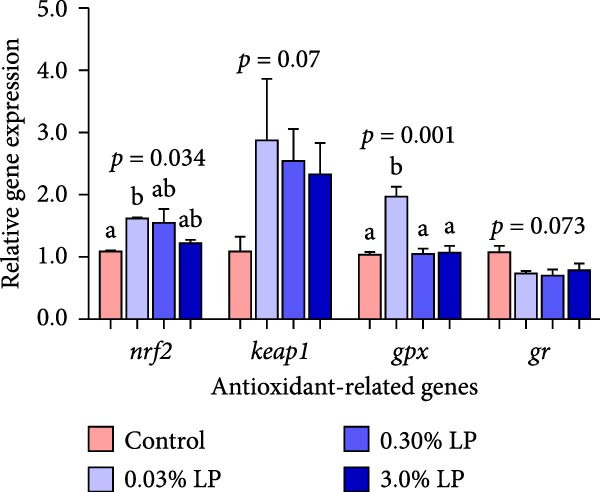
(A)

**Figure:**
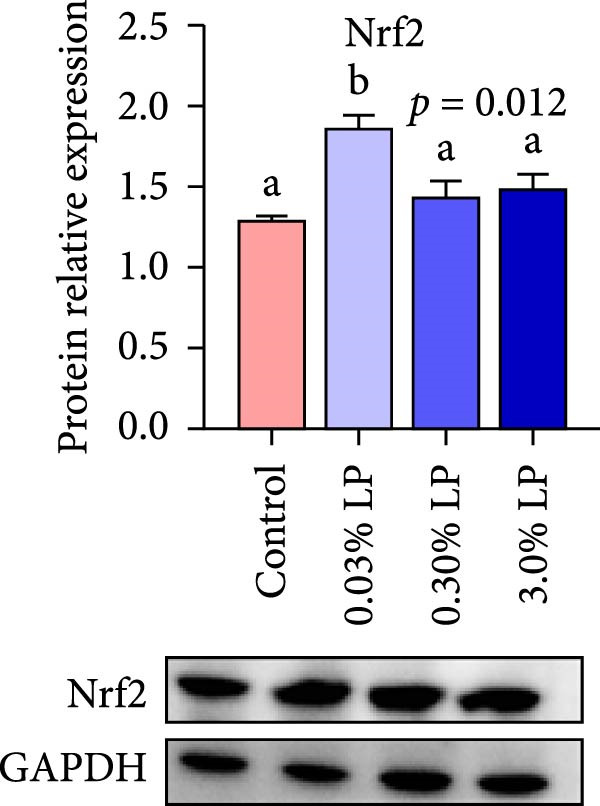
(B)

**Figure:**
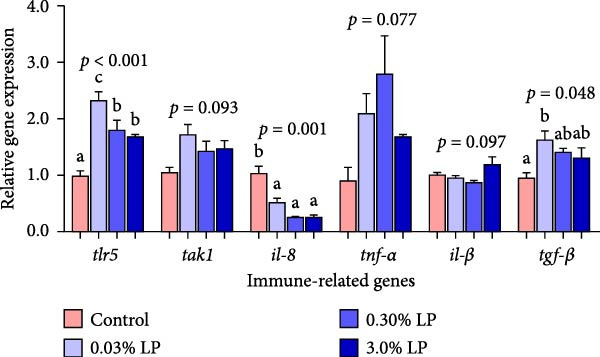
(C)

### 3.4. Immune‐Related Indices in Liver

The effects of dietary supplementation of LP N1115 on the hepatic immune‐related physiological and molecular indices in sturgeon are displayed in Table 5 and Figure 1C. The activity of ALP was statistically decreased by dietary 0.03% LP addition compared to control group (*p* = 0.02). Similarly, the transcriptional levels of *tlr5* and *tgf-β* were remarkably improved in the 0.03% LP group relative to the control group (*p* < 0.05). Conversely, relative mRNA expression of *il-8*, a pro‐inflammatory cytokine, was significantly down‐regulated in the probiotic‐supplemented groups when compared to control group (*p* = 0.001).

### 3.5. Survival Rate Under Ammonia Stress

**Figure 2:**
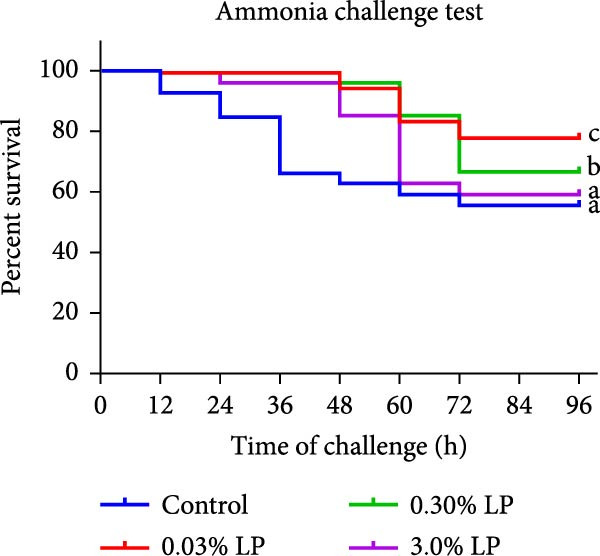
Effects of dietary supplementation of LP N1115 on survival curve in sturgeon during ammonia stress.

A 96‐h ammonia stress experiment conducted after the end of feeding trial is shown in Figure 2. Fish in the experimental groups started to die at 12 h under ammonia stress. At 96 h, the cumulative survival rate in 0.03% LP and 0.30% LP groups were significantly higher than that in control group (*p* < 0.05). The mean survival rate in control, 0.03% LP, 0.30% LP, and 3.0% LP groups were 55.56%, 77.78%, 66.67%, and 59.26%, respectively.

### 3.6. Duodenal Histology and Intestinal Microbiota

**Figure 3:**
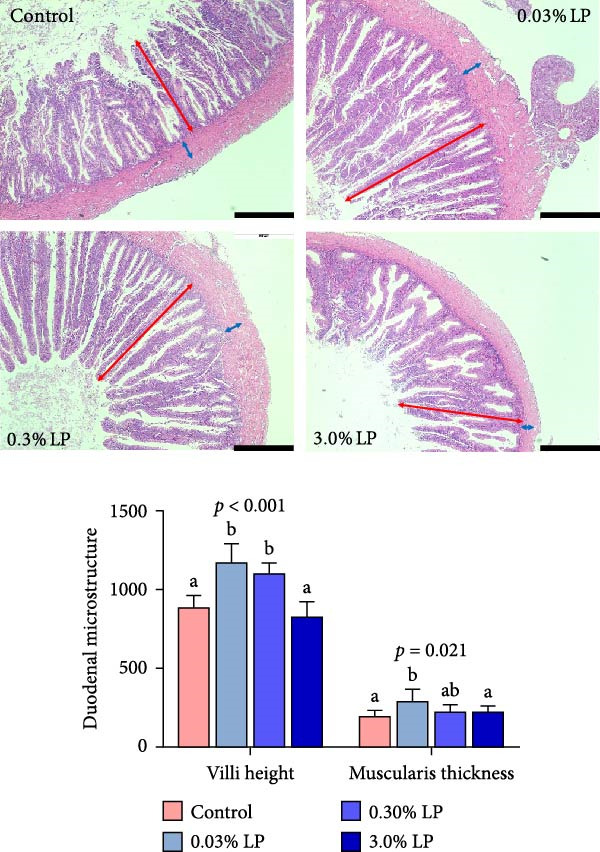
Effects of dietary addition of LP N1115 on the duodenal microstructure of sturgeon (red bidirectional arrows represent villi height, blue ones represent muscular thickness, and black scale bar = 500 µm).

The effects of varying dietary LP N1115 levels on the duodenal histology of sturgeon are illustrated in Figure 3. The villi height and muscularis thickness in duodenum tended to increase and then decrease as dietary probiotic increased, and peaked at the 0.03% LP group (*p* < 0.05).

Based on the Venn diagram presented in Figure 4A, the total OTU number of intestinal microbiotas in control, 0.03% LP, 0.30% LP, and 3.0% LP groups were 587, 563, 421, and 306, respectively. In terms of alpha diversity as shown in Figure 4B, the 3.0% LP group exhibited a significant reduction in the Sobs index, Chao index, and Ace index compared to the control group (*p* < 0.05). Additionally, the Shannon index of the 0.30% LP and 3.0% LP groups was remarkably lower than counterpart of the control group (*p* < 0.05). While the Simpson index showed an opposite trend to the Shannon index (*p* < 0.05).

Figure 4Venn diagram showing the unique and shared OTU in the intestine of sturgeon from control group and three probiotic‐supplemented groups (A). Alpha diversity as indicated by Sobs, Chao, Ace, Shanno, and Simpson indices in digestive tract of sturgeon fed four experimental diets (B). Data are expressed as mean ± SE (*n* = 3). Mean value with different letters above the columns are significantly different (*p* < 0.05).

**Figure:**
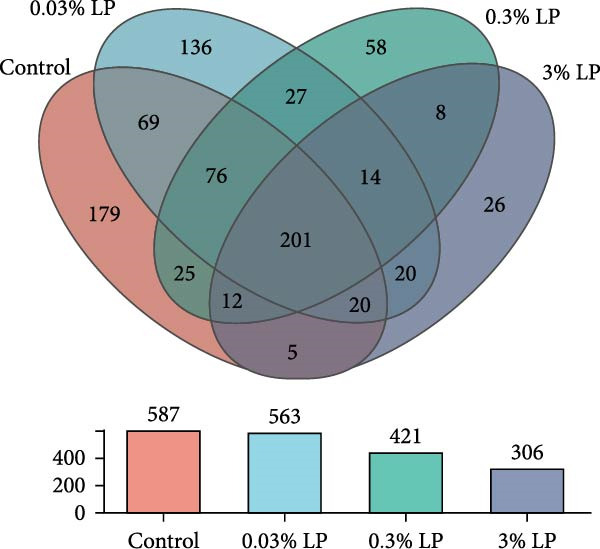
(A)

**Figure:**
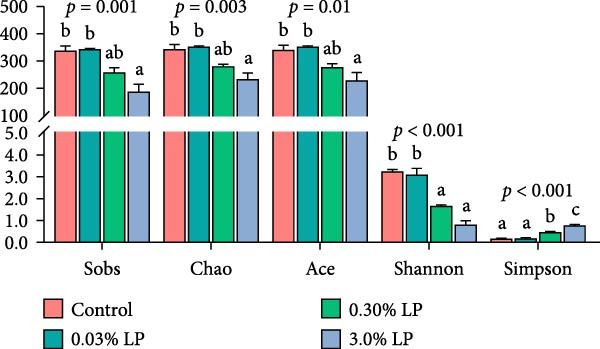
(B)

The bacterial composition at the phylum and genus levels is exhibited as a stacked bar plot as shown in Figure 5A. At the phylum level, the predominant phyla were Firmicutes, followed by Proteobacteria, Bacteroidota, Actinobacteriota, and Cyanobacteria. The average relative abundances of Firmicutes increased gradually with the increment of dietary probiotic level being, respectively, 47.92%, 60.13%, 85.00%, and 91.13% in the Control, 0.03% LP, 0.3% LP, and 3.0% LP diets. On the other hand, the relative abundance of Proteobacteria showed a decreasing trend, which were 40.25%, 22.62%, 11.16%, and 6.79%, respectively. Interestingly, the relative abundance of Actinobacteriota in 0.03% LP group (10.61%) was improved compared with control group (1.40%), 0.30% LP group (0.44%), and 3.0% LP group (1.08%).

Figure 5Average relative abundance of gut digesta at the phylum and genus levels in the hybrid sturgeon fed four experimental diets for 56 days (A). Principal coordinate analysis (PCoA) plot comparing the intestinal microbiota of the different dietary treatments with weighted unifrac distance. *R* and *p*‐values from the analysis of similarities (ANOSIM) test (B).

**Figure:**
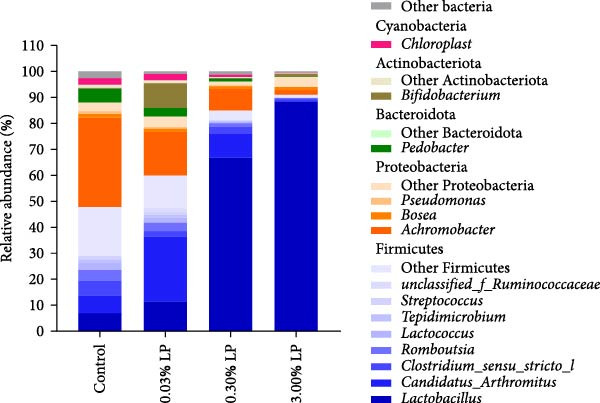
(A)

**Figure:**
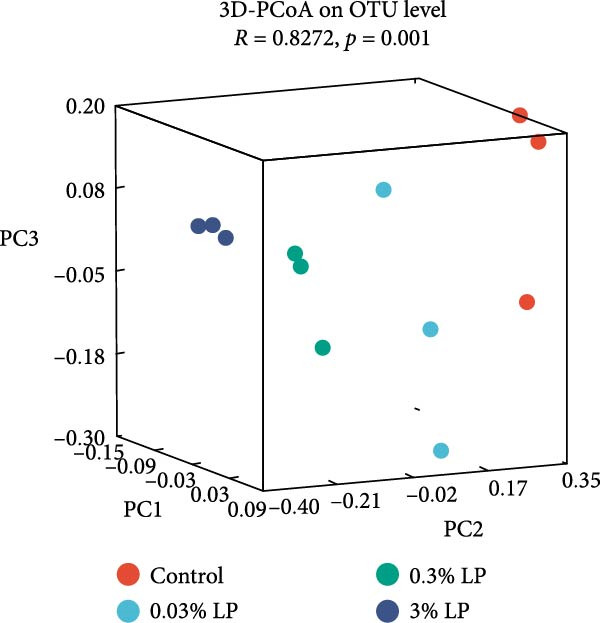
(B)

At the genus level, the relative abundance of *Lactobacillus* increased with the dietary probiotic level, with values of 7.04% (control), 11.47% (0.03% LP), 66.86% (0.3% LP), and 88.53% (3.0% LP), respectively. In addition, *Candidatus_Arthromitus* relative abundance initially increased and then decreased as the dietary probiotic level increased, and peaked at 0.03% LP group, which were 6.98% (control), 25.22% (0.03% LP), 9.17% (0.3% LP), and 0.43% (3.0% LP), respectively. More interestingly, the relative abundance of *Achromobacter* decreased as the dietary probiotic level increased, and they were 34.36% (control), 16.71% (0.03% LP), 8.31% (0.3% LP), and 1.71% (3.0% LP), respectively. In addition, the relative abundance of *Bifidobacterium* increased in 0.03% LP group (9.51%) compared with control group (0.22%). The PCoA analysis revealed distinct gut microbiota structure across groups, as evidenced by the separate cluster (*p* = 0.001) as shown in Figure 5B.

## 4. Discussion

Our research showed that growth performance (FBW, WGR, and SGR) and feed utilization of juvenile sturgeon were significantly improved by feeding 0.03% LP N1115‐supplemented diet over an 8‐week feeding trial (Table 3). In parallel, the dietary inclusion of LP I61‐27 b elevated growth performance and feed efficiency in Nile tilapia [22]. Herein, enhanced growth performance observed in sturgeon fed the 0.03% LP diet was attributed to improved feed efficiency (lower FCR) rather than feed consumption (Table 3). This improved feed efficiency was likely linked to a healthier liver, as evidenced by elevated activities of antioxidant enzymes (such as GST, T‐SOD, and TAC) and decreased levels of oxidative stress markers (MDA and H_2_O_2_) in the liver in our study (Table 5). Previous research has shown that oral administration of *Lactobacillus* could effectively mitigate oxidative stress in the host organisms [31, 32]. Moreover, the benefits of the probiotics were also reflected in duodenal health in this work, including developed villi height and a better intestine microbiota composition in sturgeon as shown in Figures 3 and 5. The healthy liver and intestine are crucial for optimizing growth performance of farmed fish.

The maintenance of the body’s oxidation‐reduction balance depends on both enzymatic and non‐enzymatic antioxidant systems [33]. In the first line of defense against reactive oxygen species (ROS), SOD catalyzes the reaction of superoxide anion to H_2_O_2_, which is then further neutralized to water by other enzymatic antioxidants, including GPx, CAT, and POD [34]. GSH is a key component of nonenzymatic antioxidants in the second line of defense and inactive H_2_O_2_ to water in the glutathione antioxidant system [35]. In the current study, sturgeon receiving the viable LP N1115 at 0.03% in the diet exhibited significantly enhanced activities of hepatic antioxidant enzymes (T‐SOD, GST) and antioxidant substance (GSH) compared to control group (Table 5). Meanwhile, a notable decrease in H_2_O_2_ content and the activities of CAT and POD was discovered in this group (Table 5). These findings suggest that more ROS was converted into water through the enhancement of SOD activity and the GSH antioxidant system in liver of fish fed with 0.03% LP diet. Moreover, the content of hepatic MDA, a marker of lipid peroxidation, was remarkably reduced in the probiotic‐supplemented groups relative to control group (Table 5). Collectively, these results indicated improved antioxidant ability and reduced oxidative stress in the liver of fish fed probiotic diets, particularly at a level of 0.03%. Similarly, the administration of LP YBJ01 significantly increased serum SOD and GPx levels while inhibiting MDA generation in mice [36]. Another strain of LP JLus66 was found to protect rats from high‐fat diet‐induced oxidative damage by increasing SOD and GPx activities and decreasing MDA concentration [37]. The enhanced antioxidant capacity observed with dietary probiotics at an optimal dosage in the current study may be attributed to several reasons. (1) Several LABs strains and their metabolites have demonstrated the ability to scavenge free radicals (H_2_O_2_ and O_2_^−^) in in vitro studies [38, 39]. (2) Probiotic supplementation optimized the intestinal microbiota structure, characterized by an increase in the proportions of beneficial microbes (*Bifidobacterium*, *Candidatus Arthromitus*, and *Lactobacillus*) and a decrease in pathogenic bacteria (*Achromobacter*) as shown in Figure 5. Excessive presence of harmful bacteria in the intestine could lead to the overproduction of ROS and induce oxidative stress [40]. It has been noted that the gut microbiota could modulate redox signaling and impact redox balance in the host [41]. Previous study has demonstrated that infection with pathogenic bacteria significantly increased hepatic and renal ROS levels on 14th day post infection in Nile tilapia [42]. (3) Certain probiotic LAB strains could activate the Nrf2 signaling pathway, a ubiquitin‐dependent pathway that responds to oxidative stress and enhances downstream antioxidant enzymes [43]. The findings of our study corroborated the significant activation effect of 0.03% LP N1115 on the transcriptional expression of *nrf2* and *gpx* in the liver of sturgeon as shown in Figure 1. (4) It has been reported that some LABs could produce GSH [44] and riboflavin [45], the latter being essential for the GSH redox cycle [46]. The present study also found that hepatic GSH content in the probiotic groups was remarkably higher than counterpart in the control group (Table 5). These results collectively indicate that dietary supplementation with probiotic LP N1115 notably enhanced the hepatic antioxidant capacity of sturgeon, particularly at a supplementation level of 0.03%.

Regarding hepatic immunity, our findings indicated that ALP and ACP activities were lowest in the 0.03% LP group, followed by 0.3% LP group (Table 5). These two phosphatases serve as key biomarkers reflecting the organism’s overall immune status [47, 48]. Similarly, dietary incorporation of another probiotic, *Lactobacillus acidophilus*, led to a significant reduction in serum ALP and ACP activities in cobia (*Rachycentron canadum*) [49]. From a pathological perspective, the decreased hepatic ALP and ACP in the sturgeon may suggest a superior health condition. To further substantiate this hypothesis, the study examined the expression of genes related to inflammatory cytokines. Not surprisingly, a notable increase in *tlr5* and *tgf-β* (an anti‐inflammatory cytokine) was observed in the 0.03% LP group, along with a significant decrease in *il-8* (a pro‐inflammatory cytokine) relative to control group as shown in Figure 1C. The attenuated immune response associated with the dietary 0.03% LP N1115 may be partially attributable to the enrichment of beneficial microbes, particularly *Bifidobacterium*, which was most abundant in this group as shown in Figure 5A. Previous research has demonstrated a relationship between increased abundance of *Bifidobacterium* and enhanced gut barrier integrity, resulting in decreased translocation of LPS into the serum and reduced immune responses [50]. Furthermore, this effect may also be associated with the harmful microorganisms, such as *Achromobacter*, which were less abundant in the probiotic‐supplemented groups as shown in Figure 5A. Prior study has suggested that an increase in pathogenic bacteria in the intestine could evoke an inflammatory response in the host [51]. Therefore, the immune responses were also modulated by appropriate addition of LP N1115 in sturgeon partly through shifting microbiota composition in the current study. It is precisely because of the improved hepatic antioxidant ability and modulated immune response in the probiotic groups, the cumulative survival rates at 96 h under ammonia stress in 0.03% LP and 0.30% LP groups were significantly higher than that in the control group as shown in Figure 2.

The intestinal microbiota is integral to the development and maturation of the host’s gut epithelium [52]. The present study indicated a significant increase in villi height and muscularis thickness in the duodenum of sturgeon fed a diet containing 0.03% LP N1115 compared to control diet as shown in Figure 3. In parallel, the application of novel LP microcapsule significantly increased villus height and villus height‐to‐crypt depth ratio in the duodenum and jejunum of broiler chickens [53]. Much deeper investigations have shown that the alteration of villus architecture in the small intestine of germ‐free mice compared with conventionally raised mice can be attributed to decreased vessel density [54] and reduced cell regeneration [55] in the gut. It has also been reviewed that the microbiota could induce substantial changes in gut morphology, encompassing villus thickness, crypt depth, and blood vessel density [52]. Alpha diversity indices are utilized to assess the richness and evenness of the intestinal microbiota. In this study, the Sobs, Chao, Ace, and Shannon indices exhibited an initial increase followed by a decrease upon probiotic supplementation, while the Simpson index displayed an opposite trend as shown in Figure 4. This phenomenon could be explained by the intermediate disturbance hypothesis from the perspective of ecology, as the gut microbiota is considered a complex ecosystem. The hypothesis states that the species diversity peaks at intermediate‐scale disturbances [56], while excessive disturbances result in a decline in species diversity. In the current study, use of 0.03% LP N1115 in the sturgeon diet is suggested as a moderate intervention on the gut microbiota, whereas higher dosages of 0.3% and 3.0% appear to be excessive interventions as shown in Figure 4.

Apart from alpha diversity, our findings indicated that the use of LP N1115 modulated the gut microbiota composition. At the phylum level, there was a rise in the abundance of Firmicutes with increasing dietary LP N1115, while the abundance of Proteobacteria displayed an opposite trend as shown in Figure 5A. It is widely acknowledged that most bacteria in Firmicutes phylum are beneficial to the host [57], while most opportunistic bacterial pathogens belong to Proteobacteria [58]. At the genus level, microbiome studies have revealed a higher abundance of beneficial bacteria (such as *Lactobacillus*, *Candidatus Arthromitus*, and *Bifidobacterium*) and lower quantities of harmful bacteria (*Achromobacter*) in fish fed 0.03% LP N1115 compared to those fed a probiotic‐free diet as shown in Figure 5A. *Lactobacillus* is known for conferring protection against invasion and colonization of opportunistic pathogens through its metabolic products such as lactic acid and bacteriocins [59]. *Candidatus Arthromitus*, and *Bifidobacterium*, play essential roles in modulating the immune barrier in the host gut [60, 61]. And the latest study has revealed that dietary supplementation of isobutyric acid significantly improved ammonia tolerance in yellow catfish (*Pelteobagrus fulvidraco*), which was closely correlated with the increased abundance of *C. Arthromitus* in the intestine [62]. Similarly, the application of *Bifidobacterium lactis* BL‐99 could alleviate intestinal inflammation and enhance intestinal function in zebrafish [63]. *Achromobacter*, an opportunistic pathogen commonly found in water and soil, could lead to infections in immunocompromised animals [64]. The mechanisms of *Lactobacillus* against intestinal pathogenic bacteria were summarized, including competitive exclusion of enteric pathogens, production of antimicrobial compounds (such as bacteriocins, organic acids, and hydrogen peroxide), reinforcement of the intestinal epithelial barrier, and modulation of the host’s immune response [65]. Therefore, the dietary supplementation of 0.03% LP N1115 optimized the gut microbiota microecosystem by increasing beneficial microbes’ populations and reducing harmful bacteria, thereby partly enhancing liver and intestinal health in sturgeon.

## 5. Conclusion

Our results indicated that the optimal incorporation of LP N1115, a novel probiotic strain derived from dairy products, significantly improved growth performance, feed efficiency, hepatic antioxidant ability partly through activating NRF2 signaling pathway, duodenal microstructure, and enhanced resistance to ammonia stress in sturgeon. Moreover, the appropriate addition of LP N1115 altered microbiota composition characterized by an enrichment of beneficial microbes and a reduction in harmful bacteria. However, a high dose (3.0% LP N1115) of this probiotic in diet resulted in a decrease in the α‐diversity of gut microbiota and had minimal impact on the liver and gut health of sturgeon, which could be partially explained by intermediate disturbance hypothesis in ecology. In summary, *L. paracasei* N1115 shows promise as a functional feed additive to enhance growth performance, liver, and intestine health in sturgeon aquaculture, with the recommended dosage being 0.03% in the diet (1.41 × 10^7^ cfu/g diet).

**Nomenclature**
LP N1115: *Lactobacillus paracasei* N1115
*nrf2*: Nuclear factor erythroid 2‐related factor 2
*keap1*: Kelch like ECH associated protein 1
*gr*: Glutathione reductase
*il-8*: Interleukin‐8
*tlr5*: Toll‐like receptors 5
*tak1*: Tgf‐β‐activated kinase 1
*tnf-α*: Tumor necrosis factor α
*il-1*β: Interleukin‐1β
*tgf-β*: Transforming growth factor‐β
IBW: Initial body weight
FR: Feeding rate
BW: Body weight
FBW: Final body weight
WGR: Weight gain rate
SGR: Specific growth rate
FCR: Feed conversion ratio
PER: Protein efficiency ratio
PRE: Protein retention efficiency
HSI: Hepatosomatic index
VSI: Viscerosomatic index
CF: Condition factor
SR: Survival rate
GSH: Glutathione
GST: Glutathione S‐transferase
GPX: Glutathione peroxidase
POD: Peroxidase
T‐SOD: Total superoxide dismutase
CAT: Catalase
MDA: Malondialdehyde
TAC: Total antioxidant capacity
ALP: Alkaline phosphatase
ACP: Acid phosphatase
SE: Standard error
PCoA: Principal coordinate analysis
one‐way ANOVA: One way analysis of variance
ROS: Reactive oxygen species
OTU: Operational taxonomic unit.

## Disclosure

An earlier version of the manuscript was published as a “preprint” in Research Place. A preprint has previously been published [66].

## Conflicts of Interest

The authors declare no conflicts of interest.

## Author Contributions

**Yanchao Yang**: methodology, software, formal analysis, investigation, writing the original draft. **Tianyu Liu**: writing the original draft, data curation. **Ling Li**: software, data curation, formal analysis. **Meng Hao**: investigation, software. **Jiarou Li**: formal analysis. **Lei Li**: data curation. **Haiyan Liu**: funding acquisition, writing – review and editing. **Baohua Zhao**: funding acquisition, writing – review and editing. **Peiyu Zhang**: conceptualisation, methodology, data curation, project administration, funding acquisition, writing – review and editing. Yanchao Yang and Tianyu Liu contributed equally to this work and are co‐first authors.

## Funding

This study was supported by the National Natural Science Foundation of China (Grant 32202946), the S&T Program of Hebei (Grant 21326703D), the Natural Science Foundation of Hebei Province (Grants C2025205014 and C2022205034), the Science and Technology Project of Hebei Education Department (Grant QN2022125), and the Hebei MATRT (Grant HBCT2024300208).

## Data Availability

The datasets used and/or analyzed during the current study are available from the corresponding author upon reasonable request.
